# The future of transcatheter aortic valve implantation is bright

**DOI:** 10.1007/s12471-023-01834-8

**Published:** 2023-11-13

**Authors:** Ronak Delewi, Michiel Voskuil

**Affiliations:** 1 Department of Cardiology, Amsterdam Cardiovascular Sciences, Amsterdam UMC, University of Amsterdam, Amsterdam, The Netherlands; 2 Department of Cardiology, University Medical Centre Utrecht, Utrecht, The Netherlands

Transcatheter aortic valve implantation (TAVI) has significantly improved treatment for patients with severe symptomatic aortic valve stenosis. TAVI has therefore been proposed as the first-line option for the majority of patients ≥ 75 years old across all risk categories [1, 2]. This minimally invasive treatment is exemplary of the ongoing development of advanced technologies in interventional cardiology and cardiothoracic surgery to improve care for patients with cardiovascular disease.

We can expect that the technologies used in TAVI will continue to evolve in the near future. These efforts will lead to better valve designs that are more durable and have enhanced haemodynamic performance [3]. Moreover, data regarding specific anatomical and clinical characteristics to guide patient-tailored treatment (choosing the right valve for the right patient) will be available. Lifetime management is already increasingly taking into account when we are planning TAVI. We are thinking about how the commissures are matching the native commissure (commissural alignment), coronary access, coronary artery flow and filling, mitigation of paravalvular leak, valve durability and future options if a valve-in-valve TAVI becomes necessary.

Innovations in procedural techniques are shifting towards more minimally invasive procedures (radial as secondary access instead of femoral, pacing over the left ventricular wire instead of extra venous access). This in turns leads to same-day admission and next-day discharge (with or without home monitoring) for patients undergoing TAVI. All these factors will lead to expansion of TAVI from older patients to younger patients with other valve abnormalities. Because technologies and indications continue to progress, we have to emphasise the importance of training cardiologists in order to allow them to (cost)-effectively make use of these possibilities. A multidisciplinary team is essential in the care of the patient planning to undergo TAVI; knowledge and the participation of cardiologists, cardiothoracic surgeons, vascular surgeons, interventional radiologists, neurologists, geriatricians, anaesthesiologists, cardiac radiologists and a strong connection with primary care are needed.

Ongoing research is vital to improve the efficacy of TAVI and to further reduce complication rates. In a small proportion of patients who undergo TAVI a thromboembolic complication may occur, leading to stroke or ‘silent’ cerebral infarction. Whether cerebral protection devices can prevent this deleterious complication still needs to be proven. In contrast, TAVI may also result in increased cardiac output and thereby enhanced cerebral blood flow in some patients. This can actually lead to an improvement in cognitive function, such as better focus and improved memory. These different cognitive and cerebral outcomes need to be systematically sorted to weigh the risks of the procedure against the benefits [1]. Lastly, in patients with severe aortic stenosis, there is often concomitant coronary artery disease requiring percutaneous coronary intervention. It needs to be investigated whether conservative treatment of coronary artery disease (treatment only if complaints remain after TAVI) is non-inferior, and therefore cost-effective, compared to an invasive strategy [4]. We are moving forward in the field of TAVI and large trials are currently being run. The future of TAVI is so bright that we will have to wear shades!
